# Assessing the role of criminality in neighbourhood safety feelings and self-reported health: results from a cross-sectional study in a Dutch municipality

**DOI:** 10.1186/s12889-019-7197-z

**Published:** 2019-07-09

**Authors:** Polina Putrik, Ludovic van Amelsvoort, Suhreta Mujakovic, Anton E. Kunst, Hans van Oers, IJmert Kant, Maria W. Jansen, Nanne K. De Vries

**Affiliations:** 1 Department of Health Promotion, School for Public Health and Primary Care (CAPHRI), Maastricht University, Peter Debyeplein 1, 6229HA Maastricht, The Netherlands; 2 Academic Collaborative Centre for Public Health Limburg, Public Health Service Southern Limburg, Heerlen, The Netherlands; 3 Department of Epidemiology, School for Public Health and Primary Care (CAPHRI), Maastricht University, Maastricht, The Netherlands; 4 Public Health, Academic Medical Center, Amsterdam, The Netherlands; 5 National Institute of Public Health and the Environment (RIVM), Bilthoven, The Netherlands; 6 Tranzo Scientific Centre for Care and Welfare, Tilburg University, Tilburg, The Netherlands; 7 Department of Health Services Research, School for Public Health and Primary Care (CAPHRI), Maastricht University, Maastricht, The Netherlands

**Keywords:** Neighbourhood health, Perceived safety, Criminality, Socio-economic factors

## Abstract

**Background:**

Neighbourhood safety has repeatedly been shown to be associated with the health and well-being of the residents. Criminality is often seen as one of the key factors affecting neighbourhood safety. However, the relationship between crime, fear of crime and feelings of safety remains underexplored.

**Methods:**

Data on socio-demographic, health and safety perceptions was extracted from the Maastricht municipality survey (the Netherlands) (*n* = 9656 adults) and merged with data on official neighbourhood crime rates from the Police Registry. Pearson correlation coefficients and multilevel logistic regression models were computed to assess the association between aspects of objective and perceived criminality, individuals’ feelings of safety and health.

**Results:**

The correlation between the *police recorded* crime and residents’ *perceptions* of the neighbourhood crime rates was weak (0.14–0.38), with the exception of violent crime (0.59), which indicates that other factors contribute to the perceptions of safety. In turn, the *perception* of higher rates of violent crime and more nuisance (on the scale 0–10) but not other types of crime or nuisance was positively associated with feeling unsafe (OR 1.27 [1.22;1.32] and 1.39 [1.33;1.46], respectively). Lower general feelings of safety at both the individual and neighbourhood level were consistently associated with worse self-rated health. Among different indicators of safety, the general feelings of safety had the most pronounced association with health, while subjective or objective measures of crime showed limited to no direct relationship with health.

**Conclusions:**

Public health policies targeting safety as a social determinant of health should consider prioritizing areas of violent crime and nuisance to improve general feelings of safety. Further research is needed to understand which factors aside from criminality are driving residents’ feelings of safety.

## Background

Living in unsafe neighbourhoods has repeatedly been shown to be associated with poor mental and physical health and lower well-being of the residents. Crime, but also fear of crime and general feelings of safety have been associated with worse self-perceived health [1, 2], higher levels of stress, more depressive symptoms and worse mental health [3–6], increased risk of coronary heart disease [7], less physical activity [8] and even adverse birth outcomes. [9] A recent study from New Zealand showed that living in an unsafe environment resulted in high cortisol (stress) hormones in pregnant women as well as poor self-rated health, with a potential impact on maternal and child health. [10] Another study from the US suggested that unsafe feelings act as a mediator between low socio-economic status and worse self-rated health. [11] Understanding the complex phenomenon of neighbourhood safety is one of the promising ways to tackle urban health issues.

In research on neighbourhood characteristics and health outcomes, aspects of the neighbourhood environment can be categorized as either objective or subjective. [12] Objective aspects are independent of an individual’s own perception. Examples include quantity and proximity to places such as health and educational facilities, parks, bars, shops, hospitals, number of accidents and crimes in the neighbourhood, etc. This information can be derived from administrative databases, official registers, researchers’ observations, and geographic information systems. Subjective neighbourhood measures are individuals’ assessments, or perceptions, of the neighbourhood living environment and may include similar domains to the objective aspects, such as perceived reachability and access to facilities, perceived criminality and safety feelings, or domains that are almost exclusively measured by individuals’ perceptions, such as social cohesion. [3, 12, 13]

Studies of neighbourhood safety often focused exclusively on the relationship between either perceived (e.g. fear of crime or general feelings of safety) *or* objective safety (i.e. recorded crime and nuisance) and health. To our knowledge, relatively few studies have explored both measures in the same study population and reflected upon a relationship between them. Some of these studies reported the independent contribution of both perceived and objective safety to health, as well as the mediating role of perceived safety in a link between objective safety and health. [4, 14] Wilson-Genderson et al. demonstrated that both actual levels of neighbourhood violence and individual perceptions of neighbourhood safety had significant effects on the depressive symptoms experienced by community-dwelling older adults. [3] Another study explored inter-relationships between objective (census-based) and subjective (resident-reported) features of the residential environment, including safety, in African-American women. [15]

However, the link between objective safety (registered crime rates), fear of crime and feelings of safety is more complex than it may intuitively seem, partly because perceived safety may cover a broader perception of the social and physical environment than just issues specifically related to crime [6, 16, 17]. Furthermore, it is possible that some types of crime contribute more to feelings of unsafety than others. To our knowledge, this has never been explored in detail. Although some evidence suggests that measures of perceived and objective safety often do not follow similar patterns, still no clear picture exists of how the objective and perceived criminality and feelings of safety are related to each other [12].

At the same time, it is of great importance for policy-makers to know how different aspects of safety relate to each other and, more importantly, to health, in addition to the notion that addressing safety has a high potential for public health. Better understanding of the underlying factors for negative safety perceptions is essential for targeted interventions to strengthen feelings of safety in the community and to improve health in the longer term.

The main objective of this study was to explore how objective and perceived neighbourhood criminality relate to each other and to feelings of safety and health, and the role of demographic and socio-economic factors in these relationships. The following research questions were set:

1. How do objective (police-recorded) and subjective (perceived) crime rates relate to feelings of safety?
2. How do objective (police-recorded) and subjective (perceived) crime rates relate to each other?
3. How do objective (police-recorded) and subjective (perceived) crime rates relate to self-rated health?

A recent study by Jackson et al. showed that individual demographic (gender in particular) and socio-economic characteristics are influential beyond the independent effect of observed social disorder cues. [18] Other research suggests that older people and persons with lower SES feel generally more vulnerable and less safe. [19, 20] Further, safety perceptions of different types of crime may differ substantially by gender, as females tend to report more unsafety feelings compared to males. [14, 21, 22] Therefore, we explored whether relationships between objective and subjective measures of safety differ by gender, age or socio-economic status.

## Methods

### Sources of data

We used data from two sources. The first source was cross-sectional survey data from the local authorities of Maastricht, the largest municipality in southern Limburg (122,488 inhabitants, 60.03 km^2^ [23]). This survey is conducted biannually by the municipal authorities among non-institutionalized inhabitants aged 18 years or over, and uses a probability sampling technique: a questionnaire is sent to a household, and the adult (≥18) person whose birthday comes first after the date on which the questionnaire was received is asked to complete it. The survey includes questions on perceived neighbourhood environment (including safety perceptions), demographic and socio-economic background, and health. We used the data from the 2010 survey (9656 respondents). The second source was the Police Registry of Crime from 2010. The registry contains the number of registered crimes (per calendar year) and the neighbourhood postal code where the crime took place.

Thirty-nine neighbourhoods (as defined by the “buurt code” by Statistics Netherlands [24]) were included in the analyses (150 to 6305 inhabitants per neighbourhood). Very small neighbourhoods with fewer than 100 residents (*n* = 3) were excluded from the sample in advance.

### Outcome variables

Individual’s feelings of safety (referred to as perceived safety) and self-rated health were the main outcomes. Survey respondents were asked whether they perceive their neighbourhood as safe generally (no vs yes), and safe at night specifically (no vs yes). Self-rated health was measured by a question, “How would you rate your health in general?”, with five answer categories further dichotomized as poor (fair or poor) vs good (excellent, very good or good).

### Independent variables

The municipality survey provided data on demographics (age and gender) and socio-economic status. Socio-economic status was assessed by level of educational achievement and income group. The six education categories mentioned in the questionnaire were classified as low education level (primary education, lower vocational education, pre-vocational secondary education), secondary education (secondary vocational education, senior general secondary education/pre-university education) or high education level (Bachelor’s degree and higher). Income group was self-reported by the respondents as ‘low’, ‘middle’ or ‘high’.

The municipality survey included questions on perceived frequency of *crime* and *nuisance (referred to as subjective crime perceptions)*. Three types of crime were recorded: thefts (bicycle thefts, thefts of outside parts of the car, damage and theft from the car, car thefts and pick-pocketing), burglaries, and violent crimes (threatening and actual violence). For nuisance, the following three types of variables were collected: traffic nuisance (frequent aggressive traffic behaviour, frequently exceeding speed limits, frequent noise from traffic, frequent smell from traffic), nuisance from neighbours (quarrels between neighbours, youth criminality, nuisance from groups of young people, nuisance from drunk people and bars and discos), and vandalism (damage to walls and buildings, damage to telephone booths). The answer categories included never, sometimes, and always. These items were grouped to match the categories of Police Registry of Crime and nuisance (number of claims (i.e. registered complaints not necessarily confirmed and prosecuted) per 100 neighbourhood inhabitants per year), namely, thefts, burglaries, violent crime, traffic nuisance, nuisance from neighbours, and vandalism *(referred to as objective crime rates).* Each individual item from the survey data was scored as 10 (always), 5 (sometimes) and 0 (never), and average of these items was assigned to each type of crime (Table 2).

### Statistical analyses

First, Pearson correlation coefficients between actual and perceived crime rates for each of the six types of crime and nuisance were computed for the general sample and separately for sub-groups organized by gender, age (< 65 years old and ≥ 65 years old), education and income. Correlation coefficients < 0.50 were assumed to be weak, between 0.50 and 0.80 were moderate, and > 0.80 were strong. [25] Next, multilevel logistic regression models were used to take into account that characteristics of respondents (modelled as fixed effects) cluster in neighbourhoods’ random effects. Two multiple logistic regression models were computed with recorded crime rates (at the neighbourhood level) and perceived crime rates (aggregated at the neighbourhood level) as variables of interest, respectively. A manual forward selection modelling approach was used (cut-off *p*-value = 0.05). Feeling unsafe, feeling unsafe at night, and health were the outcomes (all dichotomized as unsafe/safe or poor/good in case of health), and all models were adjusted for potential confounders: individual age, gender, education and income. In models with perceived crime, due to high (> 0.8) correlations between nuisance and violent crime, each of the six types of perceived crime was first modelled in a separate regression model, and then two variations of multiple regression model were computed: one excluding perceived nuisance and the second excluding perceived violent crime.

As persons who tend to have more negative perceptions of life generally or who are less healthy may be reporting both a higher perceived frequency of crime and feeling unsafe in their neighbourhood, including only individual-level measures of perceived crime and feelings of safety (from the same source) can compromise the results (same source bias). In order to separate the impact of individual perception of frequency of a particular type of crime on feeling unsafe from the impact of neighbourhood safety as a feature of the neighbourhood social environment, we computed an aggregated perception of frequency of each type of crime (i.e. an averaged measure computed from the answers of all residents of a particular neighbourhood). This aggregated measure is less sensitive to individual perceptions and may therefore be considered as leading to more objective findings. At the same time, the contribution of individual perceptions of the environment to the individual health outcome was taken into account by including a second variable which was computed as the difference between the neighbourhood mean and the assessment given by an individual. Thus, each of the six variables measuring the perception of frequency of certain types of crime was included in the model using two variables: (1) the mean of scores given by respondents from the same neighbourhood and (2) the difference between neighbourhood mean and the individual score. To explore the relationship between feelings of safety and self-rated health, feelings of safety were added to the model first at the individual level, and then as a percentage of persons in the neighbourhood who reported feeling unsafe.

To explore whether the associations between crime, unsafety feelings and health depend on the individual’s demographic or socio-economic characteristics, interactions between objectively and subjectively measured crime rates and age, gender, education and income were tested (cut-off *p*-value for interaction term was 0.10). Analyses were performed on the complete cases available for each model. Statistical package STATA 12 was used. [26]

## Results

### Study population

In total, 9656 residents of Maastricht were included in the study (response rate 25%). On average, 248 (SD 117) persons per neighbourhood returned the questionnaire (Table 6 in online Appendix). The mean age of the respondents was 55 years, and 50% was male. Some 3192 (33%) respondents were educated to a low primary level, 2284 (24%) had completed secondary education, and almost 40% (*n* = 3817) had the highest level of educational achievement. More than half of the respondents (*n* = 4911; 51%) classified themselves in the middle income group. In total, one-third of the respondents reported feeling unsafe in the neighbourhood (*n* = 3297; 34%), and two-thirds felt unsafe in the neighbourhood at night (*n* = 6428; 67%) (Table 1). In total, 13,743 crime claims were registered by police in Maastricht in 2010, or on average 14.23 per 100 residents, ranging from 3.47 in the neighbourhoods with least crime to 74.03 in the neighbourhoods with highest reported crime. Of those, 5160 (38%) claims were about thefts, 3187 (23%) concerned nuisance, 1503 (11%) burglaries, 1434 (10%) traffic complaints, 1410 (10%) acts of vandalism, and 1049 (8%) violence.

**Table 1:** Socio-demographic characteristics of the sample and perceived safety (*n* = 9656)

Variable	Mean (SD), [min-max]^a^ N(%)^b^
Age	55.2 (15.8) [18–98]
*Missing n*	*152 (1.5%)*
Gender	
Male	4783 (49.5%)
Female	4727 (49.0%)
*Missing n*	*146 (1.5%)*
Education	
Low	3192 (33.1%)
Secondary	2284 (23.6%)
High	3817 (39.5%)
*Missing n*	*363 (3.8%)*
Income (self-classified)	
Low income	1940 (20.1%)
Middle income	4911 (50.9%)
High income	2097 (21.7%)
*Missing n*	*743 (7.3%)*
Feel unsafe	3297 (34.1%)
*Missing n*	*452 (4.7%)*
Feel unsafe at night	6428 (66.6%)
*Missing n*	*339 (3.5%)*
Self-rated health	
Good	7272 (75.3%)
Poor	2152 (22.3%)
*Missing n*	232 (2.4%)

^a^for continuous variables

^b^for categorical variables

Substantial differences were found in objective and perceived criminality between neighbourhoods. In the crime registry, the most frequent crime was burglary (5.19 per 100) of residents, followed by traffic accidents (Table 2).

**Table 2:** Neighbourhood perceived and objective criminality rates, in 2010

	Mean (SD) [min-max]
Survey data on perceived crime^a^	
Perceived frequency of thefts	3.30 (2.59) [0–10]
Perceived frequency of burglaries	3.98 (3.16) [0–10]
Perceived frequency of violent crime	1.45 (2.37) [0–10]
Perceived frequency of traffic nuisance	4.85 (3.40) [0–10]
Perceived frequency of nuisance	2.23 (2.17) [0–10]
Perceived frequency of vandalism	4.66 (3.89) [0–10]
Police registry data on crime rates	
Number of thefts per 100 residents	2.74 (1.9) [0.25–8.41]
Number of burglaries per 100 residents	5.19 (8.81) [0.39–55.42]
Number of violent crimes per 100 residents	0.93 (0.91) [0.08–5.39]
Number of traffic accidents per 100 residents	2.77 (1.6) [0.93–8.96]
Number of nuisance events per 100 residents	1.32 (0.94) [0.23–5.19]
Number of acts of vandalism events per 100 residents	1.28 (0.99) [0.17–4.52]
Total number of crimes per 100 residents	14.23 (12.39) [3.47–74.03]

^a^Perceived frequency is measured on a scale from 0 (never) to 10 (always)

### Relationship between objectively and subjectively measured neighbourhood criminality

Registered crime rates had only a weak correlation with the perceived frequency of crime, with the exception of violent crime, which showed a moderate correlation (Pearson correlation coefficient = 0.6). When the correlation analysis was repeated in subsamples of males and females separately, consistently stronger correlations for all six types of crime were observed in males, while in females five out of six correlation coefficients became weaker than in the general sample. The subgroup differences reached statistical significance for violent crime and thefts. Objective and subjective rates for violent crime had the highest correlation in both males and females compared to other types of crime, with a very strong correlation in the male subsample. No relevant correlation patterns were observed by age or socio-economic status, except for vandalism, where the correlation between actual and perceived nuisance was stronger among lower- and middle-educated respondents (Table 3).

**Table 3:** Pearson correlation coefficients between official crime rates and crime perception

	Total sample	By gender		By age		By education		
		Male	Female	≥57 years old	< 57 years old	Low	Middle	High
Thefts	0.40	0.62^a^	0.44^a^	0.46	0.41	0.36	0.45	0.38
Burglary	0.38	0.57	0.29	0.32	0.43	0.52	0.78	0.28
Violent crime	0.59	0.82^a^	0.57^a^	0.55	0.63	0.47	0.91	0.67
Traffic accidents/nuisance	0.14	0.46	−0.01	0.05	0.26	0.03	0.17	0.03
Nuisance claims	0.34	0.48	0.27	0.42	0.27	0.41	0.35	0.19
Vandalism claims	0.27	0.50	0.13	0.38	0.23	0.55^a^	0.75^a^	0.10^a^

^a^Statistically significant differences between the subgroups

### Relationship between police-recorded crime rates and feelings of unsafety and self-rated health among residents

Among registered crime rates, only violent crime and nuisance caused by neighbours remained in the final multiple regression models with both general safety feelings and safety feelings at night as the outcome (separate model for each outcome) (Table 4). One additional violent crime per 100 residents of the neighbourhood was associated with 1.24 [95% CI1.02;1.51] higher odds of feeling unsafe (and 1.28 [95%CI 1.10;1.51] of feeling unsafe at night). Every additional complaint about nuisance from neighbours was associated with 1.18 [95%CI 1.08;1.30] higher OR of feeling unsafe. Significant interactions (*p* < 0.05) between violent crime and individual’s education level were detected. Stratification revealed that in highly educated persons, the association between registered violent crimes and safety feelings is not significant, while it is more pronounced in middle- and lower-educated groups (OR 1.28 [1.06;1.54], 1.38 [1.09;1.75] and 1.22 [0.99; 1.53] for lower, middle and highly educated respondents, respectively). The remaining interactions were not significant (*p* > 0.05). None of the indicators of the registered crime was associated with the self-rated health of the residents (*p* > 0.05; data not shown).

**Table 4:** Neighbourhood objective criminality rates and feelings of safety. Results from multiple multilevel logistic regression models

	Each type of crime included in a separate model		Final multivariable model	
	Feeling unsafe	Feeling unsafe at night	Feeling unsafe	Feeling unsafe at night
	OR [95% CI]			
N in the model	8380	8478	8380	8478
Thefts	1.02 [1.01;1.02]	1.03 [1.02;1.04]	–	–
Burglaries	1.20 [1.16;1.24]	1.18 [1.14;1.23]	–	–
Violent crimes	1.45 [1.36;1.55]	1.54 [1.41;1.67]	1.24 [1.02;1.51]	1.29 [1.10;1.51]
Traffic nuisance	1.21 [1.16;1.27]	1.27 [1.19;1.34]	–	–
Nuisance caused by neighbours	1.24 [1.21;1.28]	1.23 [1.19;1.26]	1.18 [1.08;1.30]	1.18 [1.09;1.27]
Vandalism	1.46 [1.36;1.57]	1.54 [1.41;1.68]	–	–

Results from multilevel logistic regression models (individuals clustered in the neighbourhoods). Perceived safety factors are scored on the scale 0 (best) to 10 (worst); All models adjusted for age, gender, education, and income

### Relationship between perceived crime rates and feelings of unsafety and self-rated health among residents

The aggregated residents’ perceptions of frequency of all six types of crime studied were significantly associated with individual feelings of unsafety. Again, the perception of higher rates of violent crime and more nuisance from neighbours was associated with the largest odds of feeling unsafe (OR 1.27 [1.22;1.32] and 1.39 [1.33;1.46], respectively) compared to the other four types of crime and nuisance (Table 5). Significant interactions were observed between burglary and vandalism with gender: perceived frequency of burglaries and vandal acts was associated with unsafety feelings in females but not in males. ORs were 1.08 [1.00;1.17] and 0.98 [0.91;1.06] for burglaries in females and males, respectively, and 1.15 [1.04; 1.28] and 1.02 [0.91;1.13] for vandalism in females and males, respectively. The remaining interactions were not significant (*p* > 0.10).

**Table 5:** Associations between perceived criminality (by type of crime) at neighbourhood level, feeling unsafe (at night) and self-rated health

	Base model: Perception of each type of crime included in a separate model		Variation 1: Multivariable model including thefts, burglaries, violent crimes, traffic nuisance, vandalism (excluded: nuisance)		Variation 2: Multivariable model including thefts, burglaries, traffic nuisance, nuisance, vandalism (excluded: violent crimes)		Base model: Perception of each type of crime included in a separate model
	Feeling unsafe	Feeling unsafe at night	Feeling unsafe	Feeling unsafe at night	Feeling unsafe	Feeling unsafe at night	Poor health
N included in the model	between 5154 and 8026	between 5219 and 8129	4574	4626	4429	4478	between 5237 and 8216
	Feel unsafe vs feel safe OR [95% CI]						Poor vs good health OR [95% CI]
Perceived frequency of thefts	**1.84** [1.63;2.07]	**1.79** [1.61;2.00]	**1.15** [1.11;1.20]	**1.15** [1.10;1.20]	**1.13** [1.08;1.17]	**1.11** [1.07;1.16]	1.06 [0.98;1.14]
Perceived frequency of burglaries	1.22 [1.00;1.48]	1.19 [0.98;1.45]	**1.04** [1.02;1.07]	**1.05** [1.02;1.08]	**1.06** [1.03;1.09]	**1.06** [1.03;1.08]	1.07 [0.98;1.16]
Perceived frequency of violent crimes	**2.29** [2.00;2.63]	**2.09** [1.81;2.41]	**1.27** [1.22;1.32]	**1.18** [1.12;1.23]	Not included^a^	Not included^a^	**1.14** [1.04;1.26]
Perceived frequency of traffic nuisance	**1.83** [1.55;2.15]	**1.64** [1.39;1.93]	**1.13** [1.11;1.16]	**1.10** [1.07;1.12]	**1.11** [1.09;1.14]	**1.08** [1.05;1.11]	**1.15** [1.06; 1.24]
Perceived frequency of nuisance caused by neighbours	**1.91** [1.61;2.26]	**1.87** [1.63;2.16]	Not included^a^	Not included^a^	**1.39** [1.33;1.46]	**1.33** [1.26;1.41]	1.03 [0.95;1.13]
Perceived frequency of vandalism	**1.69** [1.48;1.94]	**1.52** [1.34;1.72]	**1.03** [1.01;1.05]	**1.03** [1.01;1.05]	**1.02** [1.00;1.05]	**1.02** [1.00;1.04]	1.04 [0.96;1.13]

Results from multilevel logistic regression models (individuals clustered in the neighbourhoods); Perceived safety factors are scored on the scale from 0 (best) to 10 (worst); n range 5219–8129. All models adjusted for age, gender, education, income and the difference between neighbourhood mean and individual score for each factor

^a^Frequency of violent crimes and nuisance could not be added to the model at the same time due to collinearity problem

Significant estimates (at 5% level) are in bold.

When self-rated health was the outcome, only two out of six indicators of aggregated perceived crime showed significant associations, namely, perceived frequency of violent crime (OR 1.14 [1.04; 1.26]) and perceived frequency of traffic nuisance (OR 1.15 [1.06; 1.24]) (Table 5). Residents who reported feeling unsafe in their neighbourhood also reported worse health (OR 1.73 [1.54;1.94] and OR 1.43 [1.25;1.63] for general feelings of safety and specifically at night, respectively). This effect was still significant when safety was considered at the aggregated neighbourhood level, as the percentage of persons who reported feeling unsafe: residents from neighbourhoods where more people reported feeling unsafe were more likely to report worse health (OR 1.01 [1.00;1.01] for both general safety and at night, per 1% increase in respondents in the neighbourhood reporting not feeling safe). This means in a neighbourhood where 10% more residents report feeling unsafe, there are 1.06 [95% CI 1.02;1.10] higher odds that a resident will report poor health. It was striking that the percentage of residents in the neighbourhood who reported feeling unsafe ranged from 12 to 63%. Sensitivity analyses yielded similar results to the main analyses (Tables 9, 10 and 11 in the online Appendix).

## Discussion

The objective of this study was to explore how crime and fear of crime are associated with each other and with an individual’s feelings of safety and health. With regard to our first research question (How do objective and subjective crime rates relate to feelings of safety?), we found that among *objectively* (police) *registered crime*, only violent crime and nuisance from neighbours were associated with higher odds of feeling unsafe, while police records of thefts, burglary, traffic nuisance and vandalism were not significantly related to residents’ feelings of unsafety. At the same time, *individuals’ perceptions* of frequency of any of the six *types of crime* (e.g. fear of crime) were always significantly related to feelings of safety, with violent crime and nuisance having the strongest association with unsafety feelings.

The answer to our second research question (How do objective and subjective crime rates relate to each other?) is that police-registered frequency and perceived frequency of crime showed only a moderate correlation for violent crime, and no or very weak correlations for other types of crime. Interestingly, the correlations were more pronounced in men, which might indicate that men have a more realistic perception of the actual crime situation in the neighbourhood. Lower- and middle-educated people were more aware of vandalism crimes in their neighbourhood and their awareness correlated well with the official registry, while those who were highly educated seemed not to be triggered by graffiti or limited damage to their physical environment. Vandalism is an indirect indicator of crime and disorder, which might become a trigger when people are confronted with a range of other social and safety issues that are known to cluster as the socio-economic status decreases.

With regard to our third research question (How do objective and subjective crime rates relate to self-rated health?), we found no direct association between objective crime rates and health, a limited relationship between fear of crime and health (only in case of violent crime and traffic nuisance), while worse general feelings of safety showed a consistent association with poorer health at the individual and neighbourhood levels. Of note, no substantially different findings were found in any of the analyses when feelings of unsafety specifically *at night* was an outcome.

Our findings that official crime rates make a modest contribution to explaining safety feelings are consistent with previous studies. [6, 27] Studies in the UK and Canada demonstrated that people consider factors like visibility and outside lighting, presence of groups of young people and ethnic minorities on the street, media reporting of crime, etc. in their judgement about their feelings of safety. [17, 28] In the Netherlands, data from other sources also indirectly support our findings, indicating that whereas actual crime rates are falling, feelings of unsafety remain stable. [29] Apparently, factors aside from crime ultimately define how neighbourhood residents perceive safety.

Furthermore, we observed a clear gender difference in the relation between objective and perceived crime frequency. Females reported higher rates of all other types of crime, and these correlated poorly with the objective numbers, in particular for violent crime and thefts. Furthermore, similar perceptions of the frequency of burglaries and acts of vandalism translated into stronger unsafety feelings in females, but not in males. A recent study by Lovasi et al. also observed that “dishonesty crime” (i.e. crime involving destruction of property) has been associated with unsafety feelings only among females. Unlike our results, Lovasi et al. didn’t find such a pattern in case of burglaries, possibly because they were analyzed together with thefts (‘property crime’), while we separated them in our study. [14] Females feel more vulnerable and are apparently more sensitive to indirect indicators of crime such as vandalism and are more anxious about burglaries. These findings suggest a strong gender component in safety feelings that should be considered by local policy-makers when designing and implementing initiatives to address issues around safety. Female perspectives on safety should not be neglected and overlooked in more generalist approaches to neighbourhood policies.

We have further demonstrated that objective crime rates have no direct relationship to self-rated health, and perceived crime has only a limited relationship to health, while more general feelings of safety have a strong association with self-rated health, and this finding is consistent with previous publications in the field. [1, 3, 30] Objective crime is a statistic which essentially covers only some of the safety and crime issues in the neighbourhood. As such, it is not surprising that people are more sensitive to what they think is happening as opposed to what has been on the police radar. This suggests that policies purely targeted at amending criminality are not necessarily expected to achieve noticeable improvements in community health outcomes, while targeting safety feelings has a higher potential for public health. In this context, it is worthwhile mentioning recent work by Browning et al. focused on ecological (“eco-”) networks – networks linking households within neighbourhoods through shared activity locations. They observed that the intensity of such networks is inversely correlated with neighbourhood crime and thus offers promising opportunities for neighbourhood policies. [31] Future research is warranted to unravel factors aside from criminality in order to improve safety feelings. A mix of qualitative and quantitative methods will likely be required to go beyond the observed association and obtain insights into the ways that an individual’s perception around neighbourhood safety is formed.

The strength and novelty of our study consist in exploring the relationships between objective and perceived safety and health in greater detail, separating the specific types of crime and nuisance. While an earlier study claimed that addressing objectively measured criminality may not have a strong effect on improving safety perceptions and health [6], our study reveals that registered criminality is positively related to feelings of safety and unsafety only for certain types of crime (violent crime and nuisance), and thus interventions to lower these specific crime rates may have a higher potential impact on health via feelings of safety. At the same time, other types of objectively recorded crime were shown to be unrelated to the population’s safety feelings. While addressing them in public health policies may not bring the desired effect, they remain important for other spheres of social and community life.

Our study also has several notable limitations. First of all, the low response to the survey (25%) may have affected the findings, which is a common limitation to population survey data. Our sample was comparable to the general Maastricht population in terms of age, gender, and education, although the number of highly educated respondents was slightly higher (Table 12 in the online Appendix). Second, while there was a risk of confounding by unmeasured individual variables (e.g. ethnicity or cultural), we did adjust our models for important individual demographic and socio-economic variables, namely age, gender, education and income, as well as population density. Last but not least, issues related to defining the neighbourhood boundaries and the fact that respondents may not refer to the same area in their responses continuously hinder the efforts to measure the area-level effects on health. [1, 32–35]

In conclusion, our results suggest that individual perceptions of crime correlate poorly with officially recorded crime, and only violent crime and nuisance affect safety feelings. Among different indicators of safety, only general feelings of safety were positively related to health, while objective or subjective measures of crime showed little or no direct relationship to health. Public health policies targeting safety as a social determinant of health should consider prioritizing areas of violent crime and nuisance to improve general feelings of safety.

## Data Availability

Datasets may be shared after the approval of the data owners.

## Appendix

**Table 6:** Number of respondents per neighborhood

Neighbourhood	Number of respondents
Binnenstad	127
Jekerkwartier	88
Kommelkwartier	148
Statenkwartier	196
Boschstraatkwartier	143
St. Maartenspoort	158
Wyck	438
Villapark	391
Jekerdal	153
Biesland	239
Campagne	191
Wolder	234
St. Pieter	49
Brusselsepoort	410
Mariaberg	262
Belfort	401
Pottenberg	211
Malpertuis	208
Caberg	223
Oud Caberg	249
Malberg	285
Dousberg-Hazendans	225
Daalhof	445
Boschpoort	207
Frontenkwartier	24
Wyckerpoort	267
Heugemerveld	256
Wittevrouwenveld	258
Nazareth	222
Limmel	143
Scharn	488
Amby	452
Borgharen	216
Itteren	114
Randwyck	293
Heugem	358
Heer	419
De Heeg	364
Vroendaal	101
Total	9,656

**Table 7:** Associations between objective crime measures and self-perceived health. Results from multiple logistic regression models, each crime measure in a separate model

n=8,591	Base model:	Sensitivity analyses Models additionally adjusted for neighborhood population density
	Poor vs good health OR [95% CI]	
Thefts	**0.98** [0.97;0.99]	**0.98** [0.97; 0.99]
Burglaries	0.97 [0.91;1.02]	0.97 [0.92; 1.03]
Violent crimes	0.92 [0.83;1.03]	0.90 [0.81; 1.01]
Traffic nuisance	0.92 [0.84;1.00]	0.91 [0.83 ;1.00]
Nuisance by neighbors	1.00 [0.96;1.05]	0.99 [0.95; 1.05]
Vandalism	0.91 [0.81;1.02]	0.89 [0.79; 1.01]

Results from multilevel logistic regression models (individuals clustered in the neighborhoods); All models adjusted for age, gender, education, income

Significant estimates (at 5% level) are in bold

Significant estimates (at 5% level) are in bold.

**Table 8:** Pearson correlation coefficients between perceptions of different types of crime

Type of crime	Thefts	Burglary	Violent crime	Traffic accidents/nuisance	Nuisance claims	Vandalism claims
Thefts	1.00					
Burglary	0.47	1.00				
Violent crime	0.59	0.37	1.00			
Traffic accidents/nuisance	0.43	0.28	0.39	1.00		
Nuisance claims	0.61	0.31	0.69	0.47	1.00	
Vandalism claims	0.49	0.27	0.33	0.29	0.38	1.00

**Table 9:** Sensitivity analyses 1: Associations between perceived criminality (by type of crime) at neighborhood level, feeling unsafe (at night) and self-rated health. Models without correction for individual difference from the aggregated score on crime perception

	Base model: Perception of each type of crime included in a separate model		Variation 1: Multivariable model including thefts, burglaries, violent crimes, traffic nuisance, vandalism (excluded: nuisance)		Variation 2: Multivariable model including thefts, burglaries, traffic nuisance, nuisance vandalism (excluded: violent crimes)		Base model: Perception of each type of crime included in a separate model
	Feeling unsafe	Feeling unsafe at night	Feeling unsafe	Feeling unsafe at night	Feeling unsafe	Feeling unsafe at night	Poor health
N included in the model	8,380	8,478	4,574	4,626	4,429	4,478	8,591
	Feel unsafe vs feel safe OR [95% CI]						Poor vs good health OR [95% CI]
Perceived frequency of thefts	**1.62** [1.47;1.78]	**1.56** [1.43;1.70]	**1.15** [1.11;1.20]	**1.15** [1.10;1.20]	**1.13** [1.08;1.17]	**1.11** [1.07;1.16]	**1.04 **[0.97; 1.11]
Perceived frequency of burglaries	**1.22** [1.02;1.46]	1.18 [0.99;1.41]	**1.04** [1.02;1.07]	**1.05** [1.02;1.08]	**1.06 **[1.03;1.09]	**1.06** [1.03;1.08]	**1.07** [0.99;1.15]
Perceived frequency of violent crimes	**2.00** [1.78;2.25]	**1.81** [1.59;2.06]	**1.27** [1.22;1.32]	**1.18 **[1.12;1.23]	Not included^a^	Not included^a^	**1.12** [1.03;1.22]
Perceived frequency of traffic nuisance	**1.73** [1.48; 2.01]	**1.58** [1.36;1.85]	**1.13 **[1.11;1.16]	**1.10** [1.07;1.12]	**1.11 **[1.09;1.14]	**1.08** [1.05;1.11]	**1.14 **[1.05; 1.23]
Perceived frequency of nuisance by neighbours	**1.68 **[1.48;1.90]	**1.59** [1.42;1.79]	Not included^a^	Not included^a^	**1.39** [1.33;1.46]	**1.33** [1.26;1.41]	**1.06** [0.98;1.14]
Perceived frequency of vandalism	**1.66** [1.45;1.89]	**1.48** [1.31;1.68]	**1.03** [1.01;1.05]	**1.03 **[1.01;1.05]	**1.02** [1.00 ;1.05]	**1.02 **[1.00;1.04]	**1.05** [0.98;1.13]

Results from multilevel logistic regression models (individuals clustered in the neighborhoods); Perceived safety factors are scored on the scale 0 (best) to 10 (worst). All models adjusted for age, gender, education , income

^a^Frequency of violent crimes and nuisance could not be added to the model at the same time due to collinearity problem

Significant estimates (at 5% level) are in bold

**Table 10:** Sensitivity analyses 2: Associations between perceived criminality (by type of crime) at neighborhood level, feeling unsafe (at night) and self-rated health. Models ran on complete cases only for all the covariates

	Base model: Perception of each type of crime included in a separate model		Variation 1: Multivariable model including thefts, burglaries, violent crimes, traffic nuisance, vandalism (excluded: nuisance)		Variation 2: Multivariable model including thefts, burglaries, traffic nuisance, nuisance vandalism (excluded: violent crimes)		Base model: Perception of each type of crime included in a separate model
	Feeling unsafe	Feeling unsafe at night	Feeling unsafe	Feeling unsafe at night	Feeling unsafe	Feeling unsafe at night	Poor health
N included in the model	4, 285 (complete cases analyses)						
	Feel unsafe vs feel safe OR [95% CI]						Poor vs good health OR [95% CI]
Perceived frequency of thefts	**1.71** [1.53;1.91]	**1. 65**[1.49;1.83]	**1.15** [1.11;1.20]	**1.14** [1.10;1.19]	**1.13** [1.09;1.18]	**1.11** [1.07;1.16]	1.02 [0.94; 1.11]
Perceived frequency of burglaries	1.28 [1.03;1.28]	1.18 [0.96;1.45]	**1.03** [1.01;1.06]	**1.05** [1.02;1.08]	**1.06** [1.03;1.09]	**1.06** [1.03;1.09]	1.04 [0.95;1.14]
Perceived frequency of violent crimes	**2.15** [1.86;2.48]	**1.96** [1.68;2.29]	**1.28** [1.23;1.34]	**1.19** [1.13;1.25]	Not included^a^	Not included^a^	1.09 [0.98;1.22]
Perceived frequency of traffic nuisance	**1.77** [1.48; 2.13]	**1.63** [1.36;1.96]	**1.14** [1.11;1.17]	**1.10** [1.08;1.13]	**1.11**[1.08;1.14]	**1.08** [1.05;1.11]	**1.10** [1.01; 1.21]
Perceived frequency of nuisance by neighbours	**1.79** [1.54;2.06]	**1.71** [1.49;1.97]	Not included^a^	Not included^a^	**1.39** [1.33;1.46]	**1.33**[1.27;1.42]	1.06 [0.97;1.16]
Perceived frequency of vandalism	**1.76** [1.51;2.05]	**1.60** [1.40;1.84]	**1.03** [1.01;1.06]	**1.03**[1.01;1.05]	**1.02**[1.00 ;1.05]	**1.02** [1.00;1.04]	1.07 [0.98;1.16]

Results from multilevel logistic regression models (individuals clustered in the neighborhoods); Perceived safety factors are scored on the scale 0 (best) to 10 (worst). All models adjusted for age, gender, education , income

^a^Frequency of violent crimes and nuisance could not be added to the model at the same time due to collinearity problem

Significant estimates (at 5% level) are in bold

**Table 11:** Sensitivity analyses 3: Associations between perceived criminality (by type of crime) at neighborhood level, feeling unsafe (at night) and self-rated health. Models additionally adjusted for population density

	Base model: Perception of each type of crime included in a separate model		Variation 1: Multivariable model including thefts, burglaries, violent crimes, traffic nuisance, vandalism (excluded: nuisance)		Variation 2: Multivariable model including thefts, burglaries, traffic nuisance, nuisance vandalism (excluded: violent crimes)		Base model: Perception of each type of crime included in a separate model
	Feeling unsafe	Feeling unsafe at night	Feeling unsafe	Feeling unsafe at night	Feeling unsafe	Feeling unsafe at night	Poor health
N included in the model	between 5,154 and 8,026	between 5,219 and 8,129	4,574	4,626	4,429	4,478	between 5,237 and 8,216
	Feel unsafe vs feel safe OR [95% CI]						Poor vs good health OR [95% CI]
Perceived frequency of thefts	**1.78** [1.53; 2.06]	**1. 70** [1.49;1.93]	**1.15** [1.11;1.20]	**1.14 **[1.10;1.19]	**1.13** [1.08;1.17]	**1.10** [1.06;1.15]	**1.04** [0.95; 1.14]
Perceived frequency of burglaries	**1.35** [1.14;1.60]	**1.30** [1.11;1.52]	**1.03** [1.01;1.06]	**1.05** [1.03;1.08]	**1.06** [1.03;1.09]	**1.06** [1.03;1.09]	**1.07** [0.99;1.17]
Perceived frequency of violent crimes	**2.16** [1.84;2.53]	**1.86 **[1.56;2.18]	**1.28** [1.23;1.33]	**1.18** [1.12;1.23]	Not included^a^	Not included^a^	**1.13 ** [1.02;1.26]
Perceived frequency of traffic nuisance	**1.67** [1.40; 1.99]	**1.44** [1.21;1.71]	**1.13** [1.11;1.16]	**1.10** [1.07;1.12]	**1.11** [1.09;1.14]	**1.08** [1.05;1.11]	**1.15** [1.05; 1.26]
Perceived frequency of nuisance by neighbours	**1.74** [1.41;2.13]	**1.67** [1.40;1.98]	Not included^a^	Not included^a^	**1.39** [1.33;1.46]	**1.33** [1.27;1.40]	1.02 [0.91;1.13]
Perceived frequency of vandalism	**1.57** [1.35;1.84]	**1.36** [1.19;1.57]	**1.03** [1.01;1.05]	**1.03** [1.01;1.05]	**1.02** [1.00 ;1.05]	**1.02** [1.00;1.04]	1.03 [0.94;1.13]

Results from multilevel logistic regression models (individuals clustered in the neighborhoods); Perceived safety factors are scored on the scale 0 (best) to 10 (worst). All models adjusted for age, gender, education , income

^a^Frequency of violent crimes and nuisance could not be added to the model at the same time due to collinearity problem

Significant estimates (at 5% level) are in bold

**Table 12:** Dutch general population composition for age, gender and education, 2010

Variable	Dutch general population, 2010
Mean age	47.3
Males, N (%)	8,203,476 (49%)
Education level:	
Low	4,781,000 (36%)
Middle	5,125,000 (38%)
High	3,446,000 (26%)

Source: CBS, 2010
